# Predicting Longitudinal Cognitive Decline and Alzheimer’s Conversion in Mild Cognitive Impairment Patients Based on Plasma Biomarkers

**DOI:** 10.3390/cells13131085

**Published:** 2024-06-22

**Authors:** Min-Koo Park, Jinhyun Ahn, Young-Ju Kim, Ji-Won Lee, Jeong-Chan Lee, Sung-Joo Hwang, Keun-Cheol Kim

**Affiliations:** 1 Department of Biological Sciences, College of Natural Sciences, Kangwon National University, Chuncheon 24341, Republic of Korea; mkparc@kangwon.ac.kr; 2 Hugenebio Institute, Bio-Innovation Park, Erom, Inc., Chuncheon 24427, Republic of Korea; jwlee@erom.co.kr (J.-W.L.); dinner89@erom.co.kr (J.-C.L.); 3 Department of Management Information Systems, College of Economics & Commerce, Jeju National University, Jeju 63243, Republic of Korea; jha@jejunu.ac.kr; 4 Department of Statistics, Division of Economics & Information Statistics, Kangwon National University, Chuncheon 24341, Republic of Korea; ykim7stat@kangwon.ac.kr; 5 Integrated Medicine Institute, Loving Care Hospital, Seongnam 463400, Republic of Korea; lovingconcern.sjh@gmail.com

**Keywords:** plasma biomarker, neuropsychological measure, linear mixed model, cognitive decline, MCI-to-AD conversion

## Abstract

The increasing burden of Alzheimer’s disease (AD) emphasizes the need for effective diagnostic and therapeutic strategies. Despite available treatments targeting amyloid beta (Aβ) plaques, disease-modifying therapies remain elusive. Early detection of mild cognitive impairment (MCI) patients at risk for AD conversion is crucial, especially with anti-Aβ therapy. While plasma biomarkers hold promise in differentiating AD from MCI, evidence on predicting cognitive decline is lacking. This study’s objectives were to evaluate whether plasma protein biomarkers could predict both cognitive decline in non-demented individuals and the conversion to AD in patients with MCI. This study was conducted as part of the Korean Longitudinal Study on Cognitive Aging and Dementia (KLOSCAD), a prospective, community-based cohort. Participants were based on plasma biomarker availability and clinical diagnosis at baseline. The study included MCI (n = 50), MCI-to-AD (n = 21), and cognitively unimpaired (CU, n = 40) participants. Baseline plasma concentrations of six proteins—total tau (tTau), phosphorylated tau at residue 181 (pTau181), amyloid beta 42 (Aβ42), amyloid beta 40 (Aβ40), neurofilament light chain (NFL), and glial fibrillary acidic protein (GFAP)—along with three derivative ratios (pTau181/tTau, Aβ42/Aβ40, pTau181/Aβ42) were analyzed to predict cognitive decline over a six-year follow-up period. Baseline protein biomarkers were stratified into tertiles (low, intermediate, and high) and analyzed using a linear mixed model (LMM) to predict longitudinal cognitive changes. In addition, Kaplan–Meier analysis was performed to discern whether protein biomarkers could predict AD conversion in the MCI subgroup. This prospective cohort study revealed that plasma NFL may predict longitudinal declines in Mini-Mental State Examination (MMSE) scores. In participants categorized as amyloid positive, the NFL biomarker demonstrated predictive performance for both MMSE and total scores of the Korean version of the Consortium to Establish a Registry for Alzheimer’s Disease Assessment Packet (CERAD-TS) longitudinally. Additionally, as a baseline predictor, GFAP exhibited a significant association with cross-sectional cognitive impairment in the CERAD-TS measure, particularly in amyloid positive participants. Kaplan–Meier curve analysis indicated predictive performance of NFL, GFAP, tTau, and Aβ42/Aβ40 on MCI-to-AD conversion. This study suggests that plasma GFAP in non-demented participants may reflect baseline cross-sectional CERAD-TS scores, a measure of global cognitive function. Conversely, plasma NFL may predict longitudinal decline in MMSE and CERAD-TS scores in participants categorized as amyloid positive. Kaplan–Meier curve analysis suggests that NFL, GFAP, tTau, and Aβ42/Aβ40 are potentially robust predictors of future AD conversion.

## 1. Introduction

By 2023, more than 55 million people worldwide are expected to have Alzheimer’s disease (AD), the leading cause of dementia. This figure is projected to increase to 82 million by 2030 and 150 million by 2050, with nearly 10 million new cases emerging annually [1,2,3]. The clinical spectrum of AD encompasses a continuum from asymptomatic stages to mild cognitive impairment (MCI), progressing to mild, moderate, or severe AD. Patients with AD among participants with MCI, referred to as prodromal AD, have a decline in cognitive function that is greater than that expected for the participant’s age and years of education, yet without impeding activities of daily living [4]. Differentiating MCI from normal aging is challenging due to subtle symptoms often misinterpreted by patients and caregivers. While patients with MCI who have underlying AD pathology may progress to mild AD dementia, the duration of this progress varies widely among older adults. It remains unclear whether and how best to predict cognitive decline, which may indicate the onset of clinical progression.

Despite the availability of anti-amyloid beta (Aβ) treatments capable of ameliorating clinical cognitive decline in early AD, there are no disease-modifying therapies. It is understood that these drugs can merely delay disease progression, and while the removal of Aβ plaques is beneficial, it does not constitute a cure for AD. Consequently, the early detection of MCI patients at elevated risk for AD conversion is imperative for optimizing therapeutic interventions, particularly in the context of anti-Aβ therapy [3,5,6,7]. Although studies proposing combinations of plasma protein biomarkers have suggested improved diagnostic accuracy for differentiating AD from MCI and cognitively unimpaired (CU) individuals [8,9], conclusive evidence regarding their performance in predicting cognitive decline in MCI and/or CU participants is lacking. This study uniquely focuses on the utility of these biomarkers in predicting cognitive decline and conversion to AD, emphasizing the dynamic aspect of cognitive changes and the potential for early intervention.

Numerous research groups have cross-validated specific plasma protein biomarkers for AD diagnosis. Aβ peptides, particularly Aβ42 and Aβ40, are pivotal to the amyloid cascade hypothesis of AD pathogenesis. The Aβ42/Aβ40 ratio is essential for AD diagnosis, as it correlates with amyloid plaque burden in the brain [10]. Tau proteins undergo abnormal phosphorylation in AD, leading to the formation of neurofibrillary tangles. Plasma pTau biomarkers, such as pTau181, pTau217, and pTau231, have shown high concordance with tau pathology in the brain [9,11,12]. Neurofilament light chain (NFL) is indicative of neurodegeneration, and elevated plasma NFL levels are associated with cognitive deficits and AD-related brain atrophy [13,14]. NFL proves valuable in tracking the progression of neurodegenerative diseases. Glial fibrillary acidic protein (GFAP) serves as an indicator of astrocyte activation and neuroinflammation. Elevated plasma GFAP levels correlate with amyloid pathology and cognitive decline [15,16]. GFAP, combined with other biomarkers like the Aβ42/Aβ40 ratio and NFL, enhances the diagnostic accuracy for AD, rendering it a promising tool for early detection and disease monitoring. Additionally, neurogranin, β-synuclein, and synaptosomal-associated protein 25 (SNAP-25) have emerged as promising candidates in AD research. While cerebrospinal fluid (CSF) neurogranin levels correlate with tau proteins and cognitive decline, indicating synaptic loss [17], plasma SNAP-25 levels are elevated in AD patients and correlate with cognitive function and cortical atrophy [18], suggesting their potential as blood-based biomarkers for synaptic damage in AD.

This study aimed to evaluate the utility of plasma proteins in predicting longitudinal cognitive decline in a cohort comprising CU, stable MCI, and MCI-to-AD converters. Plasma protein profiles were compared closely with neuropsychological measures in the KLOSCAD study. Among the 111 participants, 40 remained stable CU, 50 remained stable MCI, and 21 converted from MCI to AD. Cross-sectional baseline quantitation of proteins included tTau, pTau181, Aβ42, Aβ40, NFL, and GFAP, while longitudinal neuropsychological measures comprised Mini-Mental State Examination (MMSE) and Consortium to Establish a Registry for Alzheimer’s Disease Assessment Packet (CERAD-TS) scores.

Although plasma biomarkers with superior diagnostic accuracy for early AD have been reported [11,12,13,14,16,19,20], the predictive performance of several state-of-the-art protein biomarkers for cognitive decline has been rarely investigated. This study is based on the hypothesis that plasma protein biomarkers could predict cognitive decline in MCI patients after adjusting for age, sex, and years of education covariates.

This study may contribute to the development of a diagnostic test for early AD with improved accuracy, facilitating the differentiation of MCI patients who are at high risk of MCI-to-AD conversion from those who are not.

## 2. Materials and Methods

### 2.1. Participants

This study was performed as part of the Korean Longitudinal Study on Cognitive Aging and Dementia (KLOSCAD), a prospective, nationwide, population-based cohort study, which focused on evaluating cognitive aging and dementia in Koreans aged 58 years or older. Approval for the study was obtained from the institutional review board of Seoul National University Bundang Hospital (No. B-0912-089-010). Prior to enrollment, all participants provided voluntary informed consent after receiving an explanation of the study’s purpose and methods.

Baseline measurements were assessed from November 2010 to October 2014, with follow-up assessments conducted every other year until the end of 2020. This study includes data up to the third follow-up, excluding the plasma protein digital immunoassay. Participants were randomly selected from the KLOSCAD cohort, which included participants who completed all evaluations, including clinical diagnosis and neuropsychological assessments. Fifty participants were characterized as having MCI, 21 as AD converters, and 40 as CU.

Baseline clinical characteristics of participants were stratified by diagnosis. There were no statistically significant differences in clinical characteristics, except for low-density lipoprotein levels (Table S1).

During the baseline assessment, approximately 15 mL of peripheral blood was obtained from each participant using K_2_EDTA tubes (BD Biosciences, Franklin Lakes, NJ, USA). The samples were then centrifuged at 2000× *g* for 10 min within 2 h of collection at room temperature to minimize protein degradation. Plasma was carefully isolated without disturbing the buffy coat, aliquoted into 0.3 mL cryovial tubes, and promptly stored in liquid nitrogen at −80 °C. Plasma specimens remained unthawed prior to analysis.

### 2.2. Clinical Diagnosis

To diagnose cognitive disorders, geriatric neuropsychiatrists specializing in dementia research conducted standardized face-to-face diagnostic interviews, including physical and neurological examinations, using the Korean version of CERAD clinical assessment battery (CERAD-K) [21]. The Clinical Dementia Rating (CDR) scale was employed to assess the severity of dementia, considering the participant’s premorbid function [22]. Dementia was diagnosed based on the criteria outlined in the Diagnostic and Statistical Manual of Mental Disorders [23]. Only participants experiencing impairment in activities of daily living due to cognitive decline, such as memory, orientation, and judgment, were concluded to have dementia; those with cognitive decline stemming from physical disability or depression were excluded. Diagnosis was confirmed through a consensus panel conference comprising a geriatric psychiatrist, a clinical psychologist, and a nurse. The presence of Alzheimer’s dementia was determined in accordance with the criteria of the National Institute of Neurological and Communicative Disorders and Stroke and the Alzheimer’s Disease and Related Disorders Association [24], encompassing both possible and probable Alzheimer’s dementia. MCI diagnosis followed the consensus criteria of the International Working Group on MCI [25]. Participants failing to meet the criteria for MCI or dementia were classified as having normal cognition. The inclusion criterion was an age of 60 years or older. Participants meeting any of the following criteria were excluded: presence of other neurological disorders potentially impacting cognitive function, such as stroke or Parkinson’s disease; recent use of cognitive enhancers within the past three years; uncorrectable hearing impairment; and any physical condition hindering regular attendance.

The following potential confounders were assessed: age (in years), sex, education (in years), and the presence of the ApoE ε4 allele. The ApoE genotype is well-established to not only increase the risk of AD but also interact with other confounders such as age, sex, and years of education [26,27].

Previous studies have elucidated that a reduced Aβ42/Aβ40 ratio correlates with an increased amyloid cortical burden or a heightened risk of progressing to AD dementia over time [10,28,29,30]. Nonetheless, certain studies have failed to replicate these observations, casting doubt on the reliability of this ratio [31,32]. Nevertheless, this controversy is currently being resolved through a better understanding of the interactions of Aβ peptides within the plasma milieu and the adoption of cortical amyloid status, rather than clinical diagnosis, as the gold standard for assessing Aβ blood biomarkers [28,29,33]. Given the absence of amyloid positron emission tomography (PET) scan data in this cohort study, indicative of cortical amyloid burden, we utilized the plasma Aβ42/Aβ40 ratio as a predictor for amyloid PET status following methodologies proposed in previous studies. Receiver Operating Characteristic (ROC) analysis is frequently employed to assess diagnostic accuracy and ascertain the optimal cut-off value for biomarkers. The Youden index method is commonly utilized to determine the optimal cut-off value, defining it as the threshold that maximizes the Youden function, which is the difference between the true positive rate and false positive rate across all possible cut-point values [34]. Comparison of the Aβ42/Aβ40 ratio between stable CU and a composite group comprising stable MCI plus AD converters was conducted using ROC analysis in MedCalc (ver 22.009). The ROC curve was generated by plotting the true positive rate (sensitivity) against the false positive rate (1—specificity) at various threshold settings. Subsequently, the area under the curve (AUC) was calculated to quantify the overall discriminative capacity of the biomarker between the CU and MCI & AD converter groups. A higher AUC value indicates superior discriminatory power. The Youden index was computed to identify the optimal cut-off value, maximizing the sum of sensitivity and specificity. The findings of our study are as follows: AUC: 0.604, Youden index: 0.255, Optimal cut-off value: ≤0.0603, Sensitivity: 46.6%, and Specificity: 85.5%. These results suggest that while the Aβ42/Aβ40 ratio demonstrates some capability in distinguishing between stable CU individuals and those with stable MCI or AD converters, its performance is moderate, with better specificity than sensitivity. We then employed this optimal cut-off ratio as a predictor for amyloid PET status, resulting in the dichotomization of participants into Aβ42/Aβ40 ≤ 0.0603 (predicted as amyloid positives) or >0.0603 (predicted as amyloid negatives) in the subgroup analysis.

### 2.3. Neuropsychological Assessment

This study assessed the cognitive function of the participants using the CERAD-K. The CERAD-K includes the following subtests: J1, Verbal Fluency Test (Animal Category); J2, Modified Korean Version of Boston Naming Test; J3, Korean Version of MMSE for Dementia Screening; J4, Word List Memory Test; J5, Constructional Praxis Test; J6, Word List Recall Test; J7, Word List Recognition Test; J8, Constructional Recall Test; and J9 A/B, Trail-Making Test A and B [21]. The CERAD Total Score (CERAD-TS) was computed by summing J1–J7 to assess overall cognitive domains, as per prior research [35,36]. For the MMSE in the CERAD-K, all verbal questions were translated, except those related to reading and writing, which were almost identical to the words in the MMSE-K [37].

### 2.4. Protein Digital Immunoassays

Plasma protein biomarkers were quantitated using single-molecule array (Simoa) technology. This immunoassay employed a highly sensitive, paramagnetic microbead–based sandwich enzyme-linked immunosorbent assay. Plasma levels of NFL, GFAP, Aβ42, and Aβ40 were quantitated using the Simoa^®^ Neurology 4-Plex E assay (PN 103670, Quanterix, Billerica, MA, USA), while pTau181 levels were assessed with the Simoa^®^ pTau-181 Advantage V2.1 assay (PN 104111), and tTau levels with the Simoa^®^ Tau Advantage assay (PN 101552). Three plasma cryovial tubes (0.3 mL/tube) were obtained from the Human Resources Bank of Kangwon National University Hospital. Samples and controls were transferred to each well in 96-well Quanterix^®^ plates and measured in duplicate on a SIMOA HD-X platform (Quanterix, Billerica, MA, USA) using a two-step neat assay. In this assay, target antibody-coated paramagnetic beads were combined with the sample and biotinylated detector antibody in the same incubation. Target molecules present in the sample were captured by the antibody-coated beads and bound with the biotinylated antibody detector simultaneously. Plasma samples were measured at a 1:4 dilution and in a blinded manner. The analytical lower limit of quantification (LLOQ) was 0.400 pg/mL for NFL, 2.89 pg/mL for GFAP, 1.02 pg/mL for Aβ40, 0.378 pg/mL for Aβ42, 2.00 pg/mL for pTau181, and 0.062 pg/mL for tTau. Data were collected using the SIMOA HD-X analyzer with SIMOA HD-X software, version 3.1.2011.30002. The mean intra-assay coefficient of variation was less than 5%.

### 2.5. Statistical Analysis

Demographic differences among groups were assessed using analysis of variance with Tukey’s multiple comparisons test for continuous variables and Pearson’s chi-squared test for categorical variables. To evaluate the predictive power of six plasma protein biomarkers in relation to cognitive decline over a six-year follow-up period, we employed LMM analysis. The LMM is particularly suitable for handling longitudinal repeated measures, accounting for covariance structures introduced by imperfect timing or unbalanced data points. The LMM was fitted to model longitudinal decline in MMSE and CERAD-TS using the ‘lme4’ package in R software. This methodology enables the incorporation of both fixed and random effects, furnishing a robust framework for longitudinal data analysis. The fixed effects comprised baseline plasma protein biomarker levels (categorized into tertiles), time (years from baseline), and the interaction between biomarker levels and time. Random intercepts were included to account for inter-individual variability in baseline cognitive performance, while random slopes for time were considered to capture individual differences in cognitive trajectories over the study period. The dependent variable was the neuropsychological measure (MMSE or CERAD-TS), with plasma biomarker levels, time, and their interaction serving as predictors. Covariates (age, sex, years of education, ApoE ε4 status) were included in the model. The basic model included age, sex, and years of education. The individual biomarker model extended the basic model to include ApoE ε4 status and each plasma biomarker. The comprehensive biomarker model encompassed the basic model along with ApoE ε4, tTau, pTau181, Aβ42, Aβ40, NFL, GFAP, pTau181/tTau, Aβ42/Aβ40, and pTau181/Aβ42. Normality of model residuals was confirmed to ensure the appropriateness of the LMM modeling. To assess associations with the conversion of MCI to AD dementia, Kaplan–Meier analysis was employed using the ‘survminer’ and ‘survival’ packages in the R software and IBM SPSS version 27. All statistical analyses were conducted using R software, version 4.3.3 (R Foundation for Statistical Computing, Vienna, Austria), JMP Pro 17 (SAS Institute Inc., Cary, NC, USA), IBM SPSS version 27.0 (IBM, Inc., Armonk, NY, USA), and MedCalc version 22.009 (MedCalc software, Ostend, Belgium). Statistical significance was set at a two-tailed *p*-value of less than 0.05.

## 3. Results

### 3.1. Baseline Characteristics

As expected, the frequencies of the ApoE ε4 allele were higher in both MCI non-converters and converters compared to CU participants. Similar to the Aβ42/Aβ40 ratio, there was no statistically significant difference in predicted amyloid positivity (Youden-derived) between the groups. Plasma levels of GFAP and NFL exhibited an increase in both MCI non-converters and converters (*p* = 0.021 and *p* = 0.018, respectively, Table 1). The other four plasma biomarkers did not exhibit significant differences between the groups. Baseline and longitudinal measurements of MMSE and CERAD-TS significantly decreased in both MCI groups (*p* < 0.001 for both, as detailed in Table 1), and the interquartile range (IQR) also widened.

### 3.2. Longitudinal Cognitive Status Predictor, Plasma NFL

LMM analysis was conducted to assess the significant association of plasma protein biomarkers with cognitive decline as measured by neuropsychological assessments. To determine which plasma biomarker best predicts cognitive decline, this study compared baseline concentrations of tTau, pTau181, Aβ42, Aβ40, NFL, and GFAP, as well as their composite ratios Aβ42/Aβ40, pTau181/Aβ42, and pTau181/tTau, with cognitive scores.

**Figure 1:**
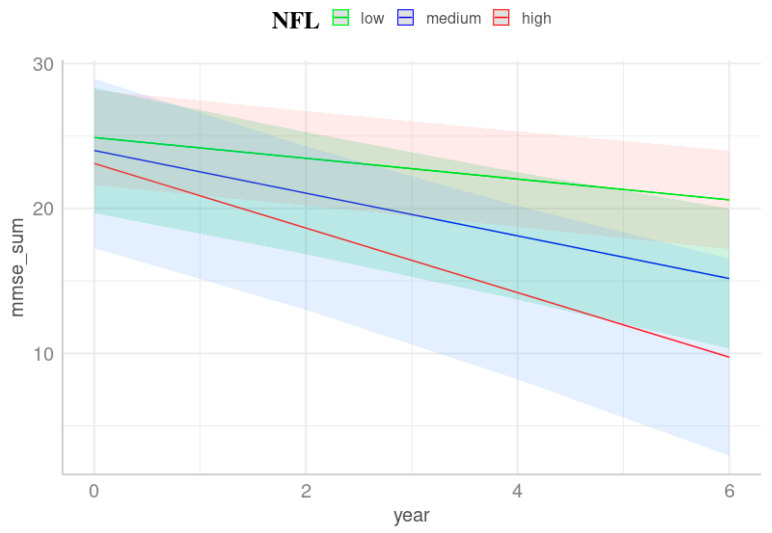
Prediction of plasma NFL on longitudinal MMSE differences. Trajectories were derived from LMM with baseline plasma NFL levels as a predictor, adjusted for age, sex, years of education, and ApoE ε4. The trajectories elucidate alterations in MMSE scores over time influenced by varying tertiles of baseline plasma NFL levels. The slope, indicative of the rate of cognitive decline, appears steeper for individuals with elevated NFL levels. The red line represents the highest tertile of NFL, while the green and blue lines represent the lowest and intermediate tertiles, respectively. Shaded areas indicate the 95% confidence intervals of the regression lines. This figure depicts mean levels within each covariate (age and years of education), with females as the reference group. The time span was capped at six years, corresponding to three follow-up assessments.

Following adjustment for the risk conferred by age, sex, ApoE ε4 allele, and years of education, LMM analysis revealed that the highest tertile of NFL can predict MMSE changes (β = −0.22, *t* = −2.28, *F* = 5.22, *p* = 0.025, Figure 1, Table 2). In other words, plasma NFL exhibited an interaction effect with the period on MMSE changes. Even without adjustment for ApoE ε4, similar predictive performance was identified (β = −0.20, *t* = −2.03, *F* = 4.13, *p* = 0.045). The other proteins, except for NFL, did not show any significant prognostic prediction for MMSE or CERAD-TS changes longitudinally. Contrary to the comprehensive model, none of the individual biomarker models exhibited significance for both MMSE and CERAD-TS changes longitudinally and cross-sectionally.

### 3.3. Baseline Biomarkers and Longitudinal Cognition Measures Based on Amyloid Positivity

**Figure 2:**
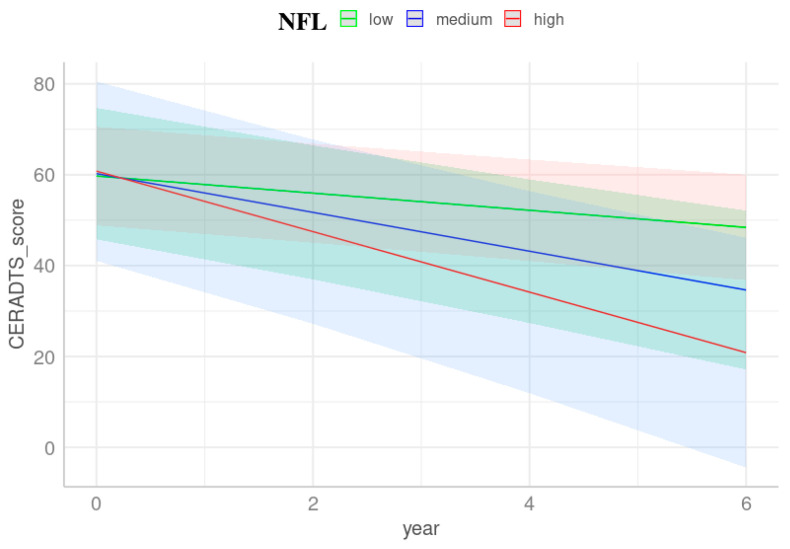
CERAD-TS trajectories stratified by NFL tertiles. Trajectories were derived from LMM with baseline plasma NFL over time as a predictor, adjusted for age, sex, and years of education. The trajectories elucidate alterations in CERAD-TS scores over time influenced by varying tertiles of baseline plasma NFL levels. The slope, indicative of the rate of cognitive decline, appears steeper for individuals with elevated NFL levels. The red line is fitted to the highest tertile of NFL, while the green and blue lines represent the lowest and intermediate tertiles, respectively. Shaded areas represent the 95% confidence intervals of the regression lines. This figure represents mean levels within each covariate (age and years of education) and females as the reference group. The time span was capped at six years, corresponding to three follow-up assessments.

Participants categorized as amyloid positives demonstrated that the highest tertile of NFL revealed an interaction effect with the period on CERAD-TS changes (β = −0.85, *t* = −1.91, *F* = 4.33, *p* = 0.049, Figure 2, Table 3) and furthermore predicted the MMSE changes longitudinally (β = −0.29, *t* = −2.00, *F* = 3.97, *p* = 0.047, Table 3). This indicates that plasma NFL biomarkers exhibited predictive performance for both MMSE and CERAD-TS trajectories. From this, it can be speculated that over six years of follow-up, participants with unimpaired cognition may delay the decline in performance on cognition measures, while those predicted as amyloid positive with the highest tertile of plasma NFL may experience a faster decline, reflecting steady worsening. Participants categorized as amyloid negatives did not show any significant predictive performance in either measure.

**Figure 3:**
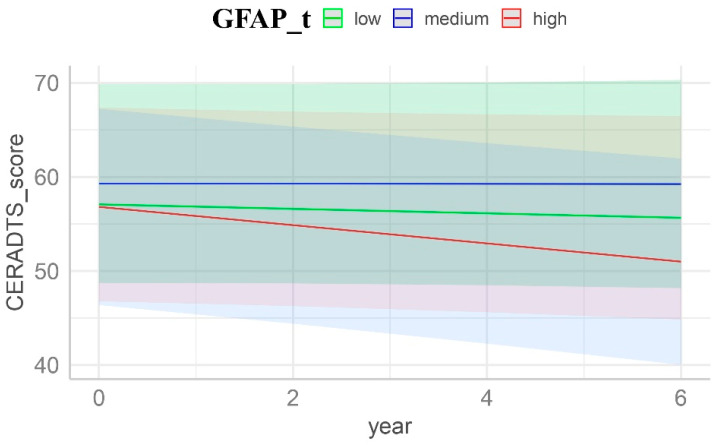
CERAD-TS trajectories stratified by GFAP tertiles. Trajectories were derived from LMM with plasma GFAP over time as a predictor, adjusted for age, sex, and years of education at baseline. The y-intercept diminishes for higher GFAP levels, indicating a lower baseline cognitive function. The red line represents the highest tertile of GFAP, while the green and blue lines represent the lowest and intermediate tertiles, respectively. Shaded areas represent the 95% confidence intervals of the regression lines. This figure represents mean levels within each covariate (age and years of education), with females as the reference group. Time was capped at six years, corresponding to three follow-up assessments.

Participants categorized as amyloid positives also revealed that the highest tertiles of GFAP (β = 6.96, *t* = 2.08, *F* = 4.33, *p* = 0.042, Figure 3, Table 3) were significantly associated with baseline cognitive scores in CERAD-TS, after adjusting for the risk conferred by age, sex, and years of education. This implies that plasma GFAP biomarkers can discriminate the cross-sectional differences in neuropsychological scores of CERAD-TS. Plasma GFAP biomarkers exhibited no significance in association with baseline MMSE scores. Participants categorized as amyloid negatives did not show any significant predictive performance in either measure.

### 3.4. Prediction of MCI-to-AD Conversion

We subsequently assessed whether plasma protein biomarkers were significantly associated with the conversion of MCI to AD. In total, 71 participants from the KLOSCAD cohort underwent evaluation for AD conversion at the six-year follow-up. Among them, 21 participants converted to AD dementia. The initial event was the diagnosis of MCI, and the endpoint event was considered the conversion from MCI to AD. For AD converters, this was defined as the time point from the baseline assessment to the diagnosis of AD. For non-converter participants who were censored at the last follow-up, the survival time was six years.

**Figure 4:**
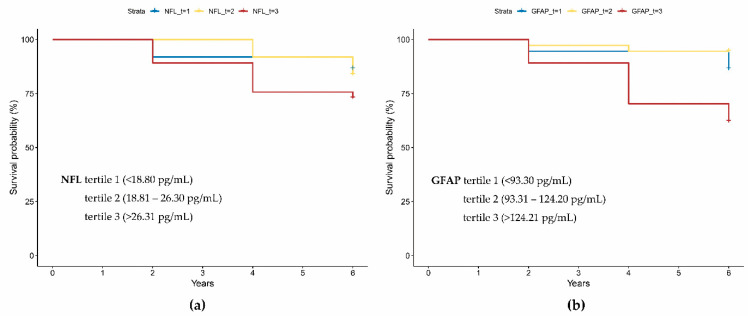

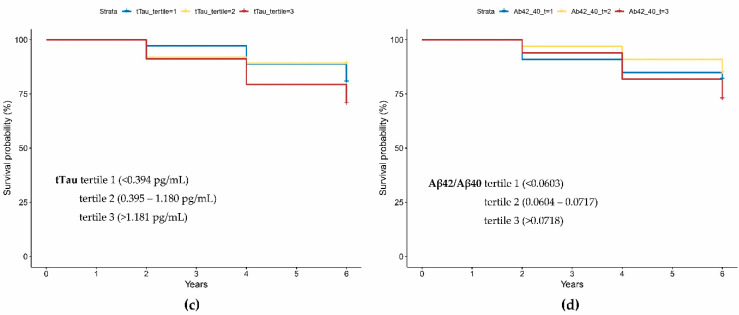
Kaplan–Meier survival curves among MCI-to-AD converter participants. Plasma concentrations (pg/mL) for each plasma protein and derivative ratio tertile are denoted in each figure inset: (**a**) NFL; (**b**) GFAP; (**c**) tTau; (**d**) Aβ42/Aβ40. The *p*-value was derived from a log-rank test. Year ‘0’ is defined as the date of the baseline diagnosis assessment. Tick marks represent participants who were AD conversion-free at the last follow-up or were censored at that time point.

In the Kaplan–Meier curve analysis implementing the log-rank test, the highest tertile of baseline NFL showed an association with MCI-to-AD conversion (χ^2^ = 10.99, df = 2, *p* = 0.004, Figure 4a). The majority of MCI patients with the lowest or intermediate tertile of NFL in their plasma remained MCI over six years. Meanwhile, those in the highest tertile of NFL revealed MCI-to-AD conversion at progressively faster rates. The highest tertile of GFAP and tTau also demonstrated predictive performance for MCI-to-AD conversion (χ^2^ = 56.62, df = 2, *p* < 0.001 and χ^2^ = 14.72, df = 2, *p* = <0.001, Figure 4b,c, respectively). In the case of the Aβ42/Aβ40 ratio, the highest tertile indicated the same predictive capability for AD conversion (χ^2^ = 6.26, df = 2, *p* = 0.044, Figure 4d).

Conversely, plasma pTau181, Aβ42, and Aβ40 did not reveal any significance in relation to the conversion of MCI to AD in this Korean cohort study.

## 4. Discussion

The ATN biomarker classification, encompassing plasma biomarkers of Aβ (A), tau (T), and neurodegeneration (N), is recommended for AD diagnosis [38]. We propose that the A category indicates biomarkers of the Aβ pathway, such as Aβ42 and the Aβ42/Aβ40 ratio. The Aβ42/Aβ40 ratio, which decreases with brain amyloidosis, has been utilized to classify AD patients. However, there is a debate, as Aβ is also produced in platelets and peripheral tissues, not only in the brain, which hinders the robust classification of Aβ positivity. Indeed, a previous study showed that the Aβ42/Aβ40 ratio was only about 10% lower in individuals without Aβ deposition in the brain [29]. The other core AD biomarker, T, comprises biomarkers of tauopathy (e.g., tTau, pTau217, pTau231), in which phosphorylated tau proteins increase with tau pathology. Ultrasensitive methods for detecting these phosphorylated tau biomarkers have enabled them to exhibit high concordance with AD pathology and thus be clinically useful as AD-specific biomarkers [9,11,12]. The A and T core biomarkers are considered standalone tests for AD diagnosis. The ratio of CSF Aβ42/Aβ40 and plasma pTau181 correlates with Aβ-PET and tau-PET findings, respectively, and can discriminate AD dementia from CU controls and non-AD neurodegenerative diseases [9,10,39].

Following the addition of N and I categories, the ATNI classification is typically considered a useful framework for staging and prognosis of AD. The ‘N’ category signifies biomarkers of neurodegeneration, which includes NFL in the fluid analyte. NFL protein has been evaluated as a neurodegeneration biomarker, with evidence suggesting that it is modestly elevated in AD and can predict both cognitive decline and the rate of neurodegeneration or neuronal injury [13,14]. The ‘I’ category indicates biomarkers of inflammation, such as GFAP. GFAP, an astrocyte protein, accurately discriminated between amyloid PET-positive and -negative cognitively normal individuals. It may serve as a reliable biomarker of neuroinflammation, neuropathology-associated astrocyte reactivity, and glial activation [15,16].

Initially, we hypothesized that plasma protein biomarkers, previously well-proven in AD progression, would be able to predict both cognitive decline and conversion to AD in MCI patients. We viewed the cognition status and prognosis across the AD progression through this ATNI framework, as researchers have also proposed that neurodegeneration can have many different causes and is not specific to AD. Therefore, N category biomarkers are not necessary for diagnosis but have been suggested to provide pathological staging information and predictive value [40]. We aimed to compare different ATNI biomarkers with each other and determine whether they have predictive performance for cognitive decline and progression to AD dementia.

Our results suggest that the ATNI framework (including Aβ42/Aβ40 ratio, tTau, NFL, and GFAP) can significantly predict AD conversion (Figure 4). This indicates that plasma ATNI biomarkers could serve as standalone predictor(s) for cognitive score prognosis. Meanwhile, other plasma biomarkers previously associated with early AD pathology did not demonstrate prognostic performance in AD conversion. We speculate that NFL and GFAP exhibit a more direct association with cognitive decline, since neurodegeneration and neuroinflammation are deeply linked to cognitive impairment. Importantly, NFL and GFAP showed significance in the subgroup categorized as amyloid positives. Therefore, we reasonably anticipate that NFL and GFAP are likely to be significant prognostic predictors in participants with pre-existing AD pathology, primarily because these participants have a high probability of marked fluctuations in neurodegeneration, neuroinflammation, and cognitive function. Taken together, while conventional amyloid and tau pathology are valuable as AD diagnosis biomarkers, our results suggest that plasma NFL and GFAP may be useful as prognosis biomarkers for cognitive impairment.

We specifically identified plasma GFAP and NFL as predictors of cognitive decline and progression during the early AD stage in CU and MCI participants. Cognition measures steadily worsened over six years in participants with high baseline GFAP or NFL levels but not in those with low levels. This suggests that these plasma biomarkers indicate who is likely to experience progressive cognitive decline over the next six years.

Numerous previous studies support this perspective. In contrast to the Aβ42/Aβ40 ratio, baseline plasma NFL was associated with cognitive decline, as measured by MMSE along the AD continuum over three years of follow-up [41]. In another study, higher plasma NFL was associated with worse cognitive performance at baseline and accelerated cognitive decline over a ten-year follow-up, with a smaller trend for decline in global cognition [42]. Higher baseline serum GFAP was associated with trajectories of cognitive decline on MMSE and all tests assessing memory, attention, and executive functioning, whereas higher NFL levels were not [43]. Serum NFL and GFAP predicted clinical progression to MCI or AD from subjective cognitive decline, and GFAP further predicted MMSE slope longitudinally [40]. Also, elevated GFAP was associated with worse cognition, and plasma GFAP was the most predictive of future cognitive decline [44]. Additionally, Aβ was correlated with increased plasma phosphorylated tau solely in individuals positive for astrocyte reactivity, suggesting astrocyte reactivity as a pivotal upstream event linking Aβ with initial tau pathology [45]. Our findings reinforce the significance of GFAP as a biomarker for identifying CU individuals at risk for cognitive decline and AD progression. Elevated plasma GFAP levels signify both reduced baseline cognitive function and an augmented response to Aβ pathology, highlighting its crucial role in clinical trial participant selection and potential therapeutic targeting.

For comprehensive models prognostically predicting MMSE and CERAD-TS changes, the highest tertile of plasma NFL was significantly associated with faster decline over time in these neuropsychological measures. Among the individual biomarker models, only plasma GFAP showed an association with cognitive impairment in the CERAD-TS at baseline. None of the plasma biomarkers demonstrated a significant interaction with time, indicating that the rate of change over time in the MMSE and CERAD-TS differs greatly according to each plasma categorical variable. This implies, first, that the comprehensive model is a better fit for all of the plasma biomarkers, suggesting that plasma biomarkers provide better prognostic performance when used in combination than when used individually. Secondly, this could be attributed to the relatively smaller effects observed in the coefficients of the individual models compared to the comprehensive model.

For the diagnosis of AD, positron emission tomography (PET) and cerebrospinal fluid (CSF) analysis have been considered the ‘gold standard’. PET imaging is available at a limited number of tertiary referral big hospitals, and CSF measurement is invasive and not easily repeatable. These factors have resulted in poor availability and have limited their widespread use as clinical diagnostics. Recently, more accessible, less-invasive blood biomarker technology has provided sufficient evidence of the underlying AD pathology, enough to overcome the previous hurdles. It could be of great value for clinical practice and trials to investigate whether plasma ATNI biomarkers perform as well as CSF biomarkers. Our study addresses the individualized and composite predictive performance of plasma biomarkers, but it should be compared with CSF and/or neuroimaging biomarker-driven prognosis, especially at the MCI stage.

This study provides plasma biomarkers that predict cognitive decline, potentially enhancing the cost-effectiveness, scalability, and explainability of clinical trials for disease-modifying treatments of AD. Regarding the recruitment of cognitively impaired participants, a previous study showed an 80% cost savings in trial screening by substituting amyloid or tau PET with a plasma biomarker [46]. Our findings also suggest that the plasma biomarkers NFL and GFAP could serve as alternatives to PET or magnetic resonance imaging for participant recruitment in clinical trials of novel anti-Aβ therapies.

Recently approved Leqembi, a probable disease-modifying drug for AD, and others in late-stage clinical trials expected to be approved soon, are monoclonal antibody drugs against Aβ. These drugs bind with high affinity to and reduce Aβ fibrils, thus slowing cognitive decline in individuals with early AD progression. Despite breaking new ground, the therapeutic effects remain modest due to the risk of amyloid-related imaging abnormalities, which occasionally result in fatal brain bleeding and seizures. The late-stage developmental anti-Aβ drug donanemab has been reported to slow cognitive decline by up to 60% in individuals starting treatment during the early stages of the disease (MCI) [47].

Clinical studies of prodromal AD aimed at developing successful anti-Aβ therapies would benefit from enrichment strategies for individuals likely to experience cognitive decline. Accurately determining whether a patient with prodromal AD will progress to AD within six years can help identify more suitable participants to achieve maximum efficacy in anti-Aβ therapy. When treatments for preclinical AD become approved, individualized prediction of cognitive decline may guide the clinical use of therapies to provide reassurance to physicians and patients. Similarly, cognitive decline is likely in the absence of therapeutic efficacy.

This study has several limitations. One limitation is the absence of amyloid PET to validate the amyloid burden in the brains of the participants in this study. Secondly, it has a relatively small number of participants and includes only a single ethnicity. Future studies should encompass multi-ethnic cohorts to enable extrapolation to broader populations. Additionally, further validating studies are required to determine the clinical efficacy of plasma NFL and/or GFAP as less invasive and cost-saving biomarkers in predicting cognitive stage transitions longitudinally. We are committed to expanding our research to address these limitations and to validate our findings in larger, multi-ethnic cohorts. We believe that our study offers a valuable foundation for subsequent research and contributes to the ongoing efforts to develop reliable blood biomarkers for the diagnosis of MCI and the prognosis of cognitive decline in non-demented individuals.

## 5. Conclusions

This study suggests that plasma GFAP levels in non-demented participants may be indicative of cross-sectional CERAD-TS scores at the baseline. From a longitudinal perspective, plasma NFL revealed predictive performance for both MMSE and CERAD-TS trajectories in participants categorized as amyloid positives. Furthermore, NFL, GFAP, tTau, and Aβ42/Aβ40 demonstrated that they are probably robust predictors of future AD conversion.

## Figures and Tables

**Table 1:** Descriptive statistics stratified by diagnosis.

Characteristic	CU	MCI (Non-Converters)	MCI (AD Converters)	*p* Value *
	(n = 40)	(n = 50)	(n = 21)	
Age	68.0 [66.5; 70.0]	71.0 [65.0; 75.0]	74.0 [71.0; 78.0]	<0.001
Gender				0.095
- Female	20 (50.0%)	36 (72.0%)	12 (57.1%)	
- Male	20 (50.0%)	14 (28.0%)	9 (42.9%)	
Years of education	14.0 [12.0; 16.0]	6.0 [2.0; 9.0]	6.0 [0.0; 9.0]	<0.001
ApoE ε4				0.154
- absent	34 (85.0%)	39 (78.0%)	14 (66.7%)	
- present	6 (15.0%)	11 (22.0%)	7 (33.3%)	
Amyloid positivity				0.268
- positive predicted	9 (25.0%)	18 (41.9%)	6 (30.0%)	
- negative predicted	27 (75.0%)	25 (58.1%)	14 (70.0%)	
tTau (pg/mL)	0.7 [0.2; 1.2]	0.6 [0.3; 1.2]	1.1 [0.3; 1.5]	0.606
pTau181 (pg/mL)	18.9 [13.8; 25.1]	20.2 [11.5; 27.2]	19.2 [14.2; 39.4]	0.519
Aβ40 (pg/mL)	47.3 [25.5; 70.9]	33.2 [16.9; 66.2]	30.8 [11.3; 62.3]	0.541
Aβ42 (pg/mL)	3.0 [1.6; 4.0]	2.8 [1.4; 3.7]	2.2 [1.0; 4.4]	0.849
GFAP (pg/mL)	105.4 [81.9; 120.8]	102.0 [72.2; 137.1]	157.5 [105.3; 186.4]	0.021
NFL (pg/mL)	20.1 [16.2; 23.9]	24.2 [17.2; 30.9]	25.6 [19.8; 41.4]	0.018
pTau181/tTau	30.5 [15.7; 42.0]	26.4 [18.1; 49.4]	25.4 [15.8; 38.7]	0.931
Aβ42/Aβ40	0.1 [0.1; 0.1]	0.1 [0.1; 0.1]	0.1 [0.1; 0.1]	0.377
pTau181/Aβ42	7.1 [5.0; 9.5]	9.0 [5.7; 13.3]	8.9 [4.4; 16.9]	0.261
MMSE_bl	29.0 [28.0; 29.0]	24.0 [21.0; 26.0]	21.0 [19.0; 26.0]	<0.001
MMSE_2yr	29.0 [28.0; 30.0]	23.5 [21.0; 25.0]	22.0 [18.0; 25.0]	<0.001
MMSE_4yr	29.0 [27.0; 30.0]	23.0 [21.0; 25.0]	18.0 [16.0; 22.0]	<0.001
MMSE_6yr	29.0 [28.0; 29.0]	23.0 [21.0; 25.0]	17.0 [13.0; 22.0]	<0.001
CERAD-TS_bl	74.5 [70.0; 80.0]	48.0 [41.0; 54.0]	44.0 [39.0; 54.0]	<0.001
CERAD-TS_2yr	78.0 [74.5; 82.5]	47.0 [39.0; 54.0]	45.0 [34.0; 51.0]	<0.001
CERAD-TS_4yr	78.5 [73.5; 82.5]	47.0 [39.0; 55.0]	38.0 [34.0; 44.0]	<0.001
CERAD-TS_6yr	79.0 [74.0; 84.0]	45.0 [39.0; 52.0]	38.0 [27.0; 41.0]	<0.001

* Fisher’s exact test for ‘Gender’ and ‘ApoE’; Kruskal-Wallis test for all other variables. Values represent either median [interquartile range] or number (% of total). Abbreviations: CU (cognitively unimpaired), MCI (mild cognitive impairment), tTau (total tau), pTau181 (phosphorylated tau at residue 181), Aβ42 (amyloid beta 42), Aβ40 (amyloid beta 40), NFL (neurofilament light chain), GFAP (glial fibrillary acidic protein), MMSE (Mini-Mental State Exam), CERAD-TS (total scores of the Korean version of the Consortium to Establish a Registry for Alzheimer’s Disease Assessment Packet).

**Table 2:** Association of plasma biomarkers with MMSE and CERAD-TS scores.

		β Coefficient	95% CI	*t*	*p* Value
**MMSE**	***Comprehensive biomarkers model***				
	NFL×time	−0.22	−0.408–0.029	−2.28	0.025
	tTau×time	−0.01	−0.311–0.286	−0.09	0.932
	pTau181×time	0.04	−0.212–0.290	0.31	0.758
	Aβ40× time	−0.01	−0.358–0.334	−0.07	0.945
	Aβ42×time	0.14	−0.181–0.460	0.86	0.391
	GFAP×time	0.12	−0.054–0.299	1.38	0.171
	pTau181/tTau×time	0.00	−0.245–0.251	0.02	0.983
	Aβ42/Aβ40×time	0.06	−0.121–0.242	0.66	0.512
	pTau181/Aβ42×time	0.02	−0.228–0.266	0.15	0.879
**CERAD-TS**	***Comprehensive biomarkers model***				
	NFL×time	−0.31	−0.930–0.311	−0.99	0.325
	tTau×time	0.09	−0.904–1.074	0.17	0.865
	pTau181×time	−0.02	−0.858–0.808	−0.06	0.953
	Aβ40×time	−0.73	−1.876–0.413	−1.27	0.208
	Aβ42×time	0.93	−0.123–1.990	1.75	0.083
	GFAP×time	−0.03	−0.604–0.550	−0.09	0.926
	pTau181/tTau×time	0.10	−0.717–0.926	0.25	0.802
	Aβ42/Aβ40×time	−0.30	−0.900–0.301	−0.99	0.325
	pTau181/Aβ42×time	0.07	−0.745–0.889	0.17	0.862

Note: LMM was adjusted for age, sex, ApoE ε4 allele, and years of education. Protein×time denotes the time interaction effect of the protein. None of the individual biomarker models showed significance for both MMSE and CERAD-TS. Abbreviations: MMSE, Mini-Mental State Examination, CERAD-TS, total scores of the Korean version of Consortium to Establish a Registry for Alzheimer’s Disease Assessment Packet. NA, not available.

**Table 3:** Association of plasma biomarkers with MMSE and CERAD-TS scores based on amyloid positivity.

		β Coefficient	95% CI	*t*	*p* Value
**MMSE**	***Comprehensive biomarkers model***				
	NFL×time	−0.29	−0.571–0.003	−2.00	0.047
	tTau×time	−0.03	−0.567–0.507	−0.11	0.912
	pTau181×time	−0.06	−0.552–0.426	−0.26	0.799
	Aβ40×time	0.22	−0.444–0.882	0.65	0.516
	Aβ42×time	0.11	−0.425–0.639	0.40	0.692
	GFAP×time	0.05	−0.248–0.340	0.31	0.760
	pTau181/tTau×time	0.16	−0.292–0.609	0.69	0.488
	Aβ42/Aβ40×time	−1.17	−3.907–1.568	−0.84	0.401
	pTau181/Aβ42×time	0.17	−0.242–0.581	0.81	0.419
**CERAD-TS**	***Comprehensive biomarkers model***				
	NFL×time	−0.85	−7.724–0.024	−1.91	0.049
	tTau×time	0.09	−1.649–1.835	0.11	0.916
	pTau181×time	−0.25	−1.845–1.335	−0.32	0.753
	Aβ40×time	−0.04	−2.176–2.101	−0.04	0.972
	Aβ42×time	0.31	−1.396–2.019	0.36	0.720
	GFAP×time	−0.27	−1.209–0.659	−0.58	0.563
	pTau181/tTau×time	0.54	−0.923–2.005	0.73	0.468
	Aβ42/Aβ40×time	−2.29	−11.182–6.595	−0.51	0.612
	pTau181/Aβ42×time	0.22	−1.120–1.556	0.32	0.749
	***Individual biomarker model***				
	NFL×time	−0.41	−0.932–0.110	−1.56	0.121
	NFL	6.18	−0.165–12.536	1.945	0.056
	tTau×time	0.03	−0.462–0.528	0.13	0.895
	pTau181×time	−0.06	−0.579–0.451	−0.25	0.806
	Aβ40×time	−0.08	−0.576–0.420	−0.31	0.757
	Aβ42×time	0.08	−0.487–0.650	0.28	0.780
	GFAP×time	0.12	−0.236–0.480	0.67	0.500
	GFAP	6.96	0.275–13.643	2.08	0.042
	pTau181/tTau×time	0.02	−0.596–0.626	0.05	0.961
	Aβ42/Aβ40×time	−2.07	−4.476–0.343	−1.70	0.092
	pTau181/Aβ42×time	−0.110	−0.611–0.391	−0.43	0.664

Note: LMM was adjusted for age, sex, and years of education. Protein×time represents the time interaction effect of the protein. None of the individual biomarker models for MMSE showed significance. Abbreviations: MMSE, Mini-Mental State Examination, CERAD-TS, total scores of the Korean version of Consortium to Establish a Registry for Alzheimer’s Disease Assessment Packet. NA, not available.

## Data Availability

The data presented in this study are available on request to the corresponding author.

## Supplementary Materials

The following supporting information can be downloaded at: https://www.mdpi.com/article/10.3390/cells13131085/s1, Table S1: Clinical and demographic characteristics stratified by diagnosis.
