# Supplementary material for: Predicting Longitudinal Cognitive Decline and Alzheimer’s Conversion in Mild Cognitive Impairment Patients Based on Plasma Biomarkers
Source: Cells. 2024 Jun 22;13(13):1085. doi: 10.3390/cells13131085 (PMC11240497; doi:10.3390/cells13131085)
Supplement: Supplementary file 1 [file cells-13-01085-s001.zip › cells-3048301-supplementary.pdf]

Table S1. Clinical and demographic characteristics stratified by diagnosis

Characteristic	CU (n=40)	MCI (Non-converters) (n=50)	MCI (AD converters) (n=21)	*p*-value*
BMI (kg/m²)	24.1 [21.8;25.8]	23.5 [21.1;26.2]	22.9 [22.2;25.3]	0.857
Hypertension**	29 (72.5%)	34 (68.0%)	14 (66.7%)	0.294
Diabetes mellitus***	9 (22.5%)	12 (24.0%)	6 (28.6%)	0.121
Cholesterol (mg/dL)	182.4 [140.2;224.6]	184.6 [131.1;239.2]	183.1 [140.5;236.6]	0.193
LDL (mg/dL)	102.5 [65.7;163.1]	103.6 [76.1;164.4]	108.3 [78.5;176.5]	0.029
Current alcohol drinking†	3 (7.5%)	4 (8.0%)	3 (14.3%)	0.352
GDS score	5.5 [2.0;9.5]	12.0 [8.0;17.0]	9.0 [6.0;16.0]	<0.001

*Fisher's exact test was used for 'Hypertension', 'Diabetes Mellitus', and 'Current alcohol drinking'; the Kruskal-Wallis test was used for other variables. Values represent either median [interquartile range] or number (% of total). **Hypertension was diagnosed when the average systolic blood pressure was ≥140 mmHg and/or the average diastolic blood pressure was ≥90 mmHg following repeated examinations. ***Diabetes Mellitus was diagnosed at a fasting blood glucose level of ≥126 mg/dL. †Current Alcohol Drinking was indicated as 'yes' when participants consumed more than 14 units of alcohol per week. Abbreviations: CU (Cognitively Unimpaired), MCI (Mild Cognitive Impairment), BMI (Body Mass Index), LDL (Low-Density Lipoprotein), GDS (Geriatric Depression Scale).
